# Current trends in the surgical management of Dupuytren’s disease in Europe: the surgeon’s perspective

**DOI:** 10.1007/s12570-012-0091-0

**Published:** 2012-03-02

**Authors:** Lars B. Dahlin, Christopher Bainbridge, Piotr P. Szczypa, Joseph C. Cappelleri, Daniel Guérin, Robert A. Gerber

**Affiliations:** 1 Department of Clinical Sciences Malmö, Hand Surgery, Lund University, Malmö, Sweden; 2 Pulvertaft Hand Centre, Royal Derby Hospital, Uttoxeter Road, Derby, UK; 3 Medical Affairs, Pfizer Ltd, Tadworth, Surrey, UK; 4 Medicines Development Group, Pfizer Inc, Groton, CT USA; 5 A + A Healthcare Research, Lyon, France

**Keywords:** Dupuytren’s disease, Cord contracture, Fasciectomy, Fasciotomy, Percutaneous needle fasciotomy, Dermofasciectomy

## Abstract

**Introduction:**

Dupuytren’s disease (DD), commonly affecting European men, is generally treated with surgery.

**Methods:**

Orthopaedic and plastic surgeons who had been practicing for >3 and <30 years and operated on ≥5 patients with DD between September and December 2008 were surveyed in 12 European countries (Czech Republic, Denmark, Finland, France, Germany, Hungary, Italy, The Netherlands, Poland, Spain, Sweden and UK). The survey assessed procedures performed, factors influencing choice of procedure, use of physical therapy and recurrence. Descriptive statistics are reported.

**Results:**

A total of 687 surgeons participated, including 579 orthopaedic and 108 plastic surgeons; 383 (56%) were hand surgeons. About 37% of surgeons performed percutaneous needle fasciotomy (PNF), 77% fasciotomy, 95% fasciectomy and 40% dermofasciectomy (DF). Surgeons’ choice of procedure was influenced by patient preferences, age, degree of contracture and recurrent disease. The percentage of surgeons prescribing physical therapy and the mean (standard deviation [SD]) duration of therapy increased with procedure complexity: PNF = 82%, 5.2 (3.9) weeks; fasciotomy = 94%, 5.3 (3.6); fasciectomy = 97%, 6.7 (5.1); and DF = 99%, 8.5 (6.4). Using survey responses, mean (SD) estimated recurrence rates decreased and estimated time to recurrence increased with procedure complexity—PNF = 44% (27%), 17 (15) months; fasciotomy = 30% (24%), 20 (18); fasciectomy = 20% (17%), 29 (23); and DF = 20% (19%), 33 (27).

**Conclusions:**

Across Europe, patient and surgical factors influence the intention to use a surgical procedure. Fasciectomy was the most commonly performed procedure type and was associated with lower recurrence than PNF or fasciotomy.

**Electronic supplementary material:**

The online version of this article (doi:10.1007/s12570-012-0091-0) contains supplementary material, which is available to authorized users.

## Introduction

Dupuytren’s disease (DD) is often treated surgically [1, 2]. In Europe, the treating surgeons are typically orthopaedic surgeons or plastic surgeons, some of whom are specialists in hand surgery. For example, an analysis of hospital records in England found that 79% of surgical procedures for palmar fascial fibromatosis were performed by trauma and orthopaedic surgeons and 19% were performed by plastic surgeons [3].

A number of different procedures are available for the surgical treatment of DD, including percutaneous needle fasciotomy (PNF; also known as percutaneous needle aponeurotomy or needle fasciotomy), fasciotomy (subcutaneous or open), fasciectomy (also known as regional palmar fasciectomy or aponeurectomy) and dermofasciectomy (DF) [4].

In a companion article [5] published in this issue, we report the results of a patient chart review, in which surgeons reviewed the charts of patients they treated with a surgical procedure for DD. This article reports the results of the general experience of those same surgeons with surgical procedures performed for DD. Such information may be important when considering and judging the new treatment strategies available for DD [4]. Differences in surgeons’ responses by country and region are of interest and will be reported in an upcoming publication.

## Methods

The study involved a survey of surgeons who perform surgical procedures for DD.

### Participating surgeons

Orthopaedic and plastic surgeons from 12 European countries were identified. The countries were selected to take into account geographic variations between middle and northern Europe (Table 1). Surgeon specialties were selected to represent a range of surgical practice; participants included specialised hand surgeons, orthopaedic surgeons and plastic surgeons who regularly treat patients with DD. Surgeons represented a mix of public and private practices. Specific surgeons were initially identified through a variety of means, including telephone directories, internet sites and directories and hospital contacts.

**Table 1:** Countries surveyed, interview methodology and respondents by specialty

		Surgeon respondents			
Country	Methodology	Total	Orthopaedic	Plastic	Hand^a^
Czech Republic	Face to face	40	35	5	14
Denmark	Online	23	23	–	17
Finland	Online	20	20	–	12
France	Face to face	91	91	–	75
Germany	Face to face or online	90	65	25	65
Hungary	Face to face	50	35	15	27
Italy	Face to face	90	80	10	38
The Netherlands	Online	42	13	29	26
Poland	Face to face	40	40	–	14
Spain	Face to face	90	80	10	40
Sweden	Online	18	18	–	10
UK	Online	93	79	14	45
Total		687	579	108	383

^a^Hand surgeons were a subgroup of orthopaedic and plastic surgeons

To be included in the study, surgeons must have been practicing >3 years and <30 years. Surgeons were required to have treated at least five patients for DD using a surgical procedure between September and December 2008. They were also required to have used at least two of the following four procedures to treat patients with DD: PNF, fasciotomy, fasciectomy or DF.

Data collection took place between November 2009 and January 2010. Surgeons responded to a questionnaire online or during a face-to-face interview.

### Survey

The survey included questions regarding the procedures the responding surgeon performs for patients with Dupuytren’s contracture, the surgeon’s level of satisfaction with different types of procedures, follow-up care typically provided or recommended following each procedure and the typical recurrence patterns following surgery. The survey used the same definitions of surgical procedures described in the patient chart review found in the companion article [5]. The text of the survey is provided as Electronic supplementary material to this article.

### Statistical analysis

Descriptive statistics were analysed and are reported as percentages and means with standard deviations (SD).

## Results

### Demographics of surgeons

A total of 687 surgeons were interviewed, of whom 579 (84%) were orthopaedic surgeons and 108 (16%) were plastic surgeons (Table 1). Of the participating surgeons, 383 (56%) were hand surgeons, including 339 orthopaedic surgeons and 44 plastic surgeons. In all countries, some orthopaedic surgeons self-identified as hand surgeons. In Germany, The Netherlands and Sweden, some plastic surgeons also self-identified as hand surgeons.

### Characteristics of physician practice

Responding surgeons had been in practice for an average (SD) of 15.1 (7.9) years, a figure that did not differ appreciably by specialty. Of the 687 surgeons, 56% practiced only in the hospital. The remaining surgeons had mixed practices; 35% of all surgeons had a mixed practice but practiced at least half the time in the hospital, and 10% had a mixed practice but spent the majority of time in the office. Of plastic surgeons, 47% had a mixed practice, a higher rate than that of orthopaedic (33%) or hand (34%) surgeons.

Overall, surgeons estimated they saw an average (SD) of 1,612 (1,507) patients in consultation in 2008. Each responding surgeon treated an estimated average (SD) of 17.5 (19.7) patients with Dupuytren’s contracture using a surgical procedure in 2008. Orthopaedic surgeons treated an average of 16.4 (18.6) patients; plastic surgeons treated 23.3 (24.3) and hand surgeons treated 20.6 (22.9).

### Procedures performed

**Fig. 1:**
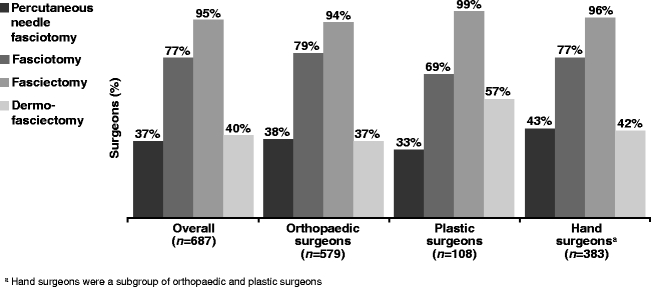
Percentage of surgeons performing each type of procedure in the past 12 months by specialty. ^a^Hand surgeons were a subgroup of orthopaedic and plastic surgeons

In a 12-month period, 255 (37%) of the 687 surgeons performed PNF for patients with DD with finger contracture, 530 (77%) performed fasciotomy, 651 (95%) performed fasciectomy and 276 (40%) performed DF (Fig. 1). These proportions were similar across surgical specialties, but plastic surgeons were more likely than orthopaedic surgeons to perform DF.

Factors influencing choice of procedure type are shown in Table 2. Surgeons’ decisions to use less aggressive procedures (PNF and fasciotomy) were influenced most strongly by older patient age, patient lifestyle factors and preferences and less severe contracture. Decisions to use more aggressive procedures (fasciectomy and DF) were influenced by more severe contracture, recurrent disease and the speed of disease progression over time.

**Table 2:** Factors influencing surgeon’s intention^a^ to use each surgical procedure

Percutaneous needle fasciotomy	Fasciotomy	Fasciectomy	Dermofasciectomy
Aged >70 years	Aged > 70 years	PIP flexion >45°	Patient presenting with recurrence
MCP flexion <20°	Patient’s job/way of life requires manual dexterity	MCP flexion >45°	PIP flexion >45°
Patient request based on aesthetic reasons	Patient request based on impairment of activities of daily living	Patient presenting with recurrence	MCP flexion >45°
Patient comorbidities or risk factors	High expectations of success following surgical procedure	Speed of progression of disease over time	Speed of progression of disease over time
Patient request based on impairment of activities of daily living	MCP flexion >45°	High expectations of success following surgical procedure	High expectations of success following surgical procedure
PIP flexion <20°	Family history of DD	Patient’s job/way of life requires manual dexterity	Family history of DD

*DD* Dupuytren’s disease, *MCP* metacarpophalangeal, *PIP* proximal interphalangeal

^a^Factors influencing intention to use a procedure were rated from 1 (“strongly decreases”) to 7 (“strongly increases”) intention to use each procedure type; factors are presented in decreasing order of importance

Among surgeons who performed each procedure, more than 65% had patients on a waiting list for each procedure type. The highest proportion was for fasciectomy; 82% of surgeons performing fasciectomy had a waiting list for the procedure. The mean estimated waiting time for all procedures was between 7 and 10 weeks, depending on the procedure type. The waiting time was similar across surgeon specialties and was longer for more invasive procedures.

### Satisfaction with procedures

On a scale of 1 (totally dissatisfied) to 7 (very satisfied), surgeons’ mean (SD) overall level of satisfaction was 5.1 (1.3) for PNF, 5.2 (1.2) for fasciotomy, 5.7 (1.0) for fasciectomy and 5.1 (1.1) for DF. Factors influencing surgeon satisfaction are shown in Table 3. Surgeons were satisfied with the low patient burden, few complications and short time to recovery associated with PNF, and they were satisfied with the high patient satisfaction and restoration of finger functionality associated with fasciotomy. However, they were dissatisfied with the high likelihood of recurrence and short time to recurrence associated with these less aggressive procedures. Surgeons were satisfied with restoration of finger functionality associated with fasciectomy and DF but were dissatisfied with the high patient burden associated with both procedures as well as the frequent complications and long time to recovery associated with DF.

**Table 3:** Factors influencing surgeon’s satisfaction with each surgical procedure

Procedure^a^	Most satisfied with	Least satisfied with
Percutaneous needle fasciotomy	Low patient burden	High likelihood of recurrence
	Few complications	Short time to recurrence
	Short time to recovery	
	High patient satisfaction	
Fasciotomy	High patient satisfaction	High likelihood of recurrence
	Restoration of finger functionality	Short time to recurrence
Fasciectomy	Restoration of finger functionality	High patient burden
	High patient satisfaction	
	Long time to recurrence	
Dermofasciectomy	Restoration of finger functionality	High patient burden
		Frequent complications
		Long time to recovery

^a^Satisfaction with each factor was rated from 1 (“not satisfied”) to 7 (“very satisfied”) for each procedure type; findings are presented qualitatively

On a scale of 1 to 7, surgeons reported their patients’ mean (SD) level of satisfaction as 5.3 (1.2) for PNF, 5.3 (1.1) for fasciotomy, 5.6 (1.0) for fasciectomy and 5.0 (1.1) for DF.

### Follow-up care

Surgeons estimated that 82% of their patients received physical therapy for the hand (including the application of a dynamic splint) following their first PNF procedure and that the mean (SD) duration of physical therapy (including use of dynamic splint) was 5.2 (3.9) weeks. Of the other procedures, 94% of patients receiving fasciotomy had physical therapy for a mean (SD) of 5.3 (3.6) weeks, 97% of patients receiving fasciectomy had therapy for a mean of 6.7 (5.1)  weeks and 99% of patients receiving DF had therapy for a mean of 8.5 (6.4) weeks. The patients of hand surgeons were slightly less likely to receive physical therapy after PNF or fasciotomy compared with patients of nonhand surgeons (PNF, 80% vs 87%; fasciotomy, 93% vs 96%). The proportion of patients receiving physical therapy after fasciectomy or DF was similar for hand and nonhand surgeons.

Most surgeons recommended that patients spend some time away from work following their surgery. The mean (SD) time surgeons recommended patients spend out of work was 2.9 (2.5) weeks following the first PNF procedure, 4.6 (2.7) weeks following fasciotomy, 5.5 (2.7) weeks following fasciectomy and 6.3 (3.2) weeks following DF. Orthopaedic surgeons recommended slightly more time out of work following each procedure type than plastic surgeons, and nonhand surgeons recommended slightly more time off than hand surgeons. Also, surgeons recommended approximately 1 week more time out of work following a reintervention procedure (compared with an initial procedure).

### Recurrence

**Fig. 2:**
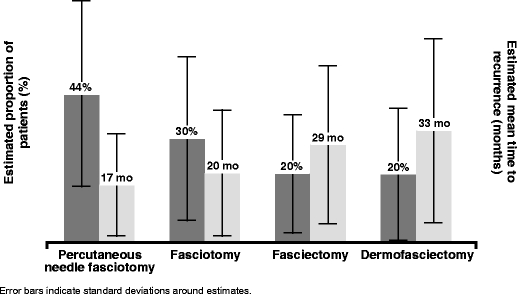
Potential for recurrence: surgeon’s report of the proportion of patients with recurrence and mean time to recurrence in the same finger following the procedure

When asked to estimate the percentage of patients who experienced recurrence of flexion in the finger that was operated, surgeons reported higher rates of recurrence and shorter time to recurrence for less aggressive procedures compared with more aggressive procedures (Fig. 2). The mean (SD) estimated recurrence rates were 44% (27%) following PNF, 30% (24%) following fasciotomy, 20% (17%) following fasciectomy and 20% (19%) following DF. The mean (SD) estimated time to recurrence was 17 (15)  months following PNF, 20 (18) months following fasciotomy, 29 (23) months following fasciectomy and 33 (27) months following DF.

## Discussion

To our knowledge, this is the only survey to date that collected, quantified and described the experiences of a large number of surgeons in the management of DD in Europe. The diverse training (orthopaedic and plastic surgeons, more than half qualified hand surgeons) and varied practice settings (hospital-based and office-based) of surgeons who participated in this study reflects a broad range of disease severity and procedures performed for DD in Europe.

In this study across Europe, fasciectomy was the surgeons’ procedure of choice for Dupuytren’s contracture, especially for patients with more advanced disease and when a more effective treatment with less likelihood of recurrence was desired. This finding is similar to that reported in the literature [3, 6]. Surgeons were more satisfied with the results of fasciectomy compared with other procedure types, and they reported that their patients were also more satisfied with fasciectomy.

Surgeons responding to this survey believed their patients who received fasciectomy and DF had lower recurrence rates and longer time to recurrence compared with patients receiving less invasive procedures, a finding that is not surprising. The rate of recurrence after fasciectomy estimated by surgeons in this study (20%) is lower than that reported in the literature (39%) [1]. This may, in part, reflect the limitations of surgeons’ recall and ability to track the progress of their patients in the years after surgery as well as the varying definitions of recurrence [2].

In some countries fasciectomy, with and without local flaps and Z-plasty, is the dominant procedure for DD. Frequency of different procedures by country may depend on the Tubiana stage of DD most commonly seen in and the surgical traditions of that country. Differences by country in the data collected for this study will be presented in a later publication.

The findings of this study demonstrate that patient factors play a role in the type of surgery recommended by surgeons. Surgeons prefer less invasive procedures for older patients and those with more comorbidities (Table 2). Surgeons also recognize the lower patient burden and lower rate of complications associated with less invasive procedures (Table 3). These issues are of particular importance in the DD patient population, which includes many older patients with complicated concomitant diseases and possibly less demand for full hand function. Diabetes often co-occurs in patients with DD [7]; this condition poses challenges to surgical treatment, including a possible higher risk of complications and delayed recovery. Even patients with impaired glucose tolerance have a higher frequency of DD; therefore, the incidence of DD can be expected to increase in the future as the global burden of diabetes increases [7].

It has been demonstrated that hand therapy after surgery results in significant improvement in total digital extension [8]. In this study, surgeons recommended physical therapy and dynamic splinting, extending for several weeks, for the majority of patients receiving surgery, with hand surgeons slightly less likely than nonhand surgeons to recommend physical therapy after PNF or fasciotomy. A limitation of this study is that duration of therapy and of splinting was not assessed independently. As stated above, surgeons reported that they recommended that patients spend several weeks out of work after most procedures. Time out of work was longer for reintervention procedures, which is not unexpected as cases involving recurrence often require more complicated surgical procedures (e.g., local flaps and split skin).

The findings regarding time in physical therapy and time out of work illustrate both the direct (surgery, physical therapy) and indirect (time out of work) costs associated with the treatment of DD. Indirect costs, including time out of work, have been found to constitute the majority of the total costs of treatment for DD and other hand injuries [9, 10]. In this study, surgeons recommended almost 3 weeks out of work following PNF and approximately 6 weeks out of work following fasciectomy and DF. In collaboration with the patient, the surgeon should consider indirect costs when choosing the surgical procedure to be performed. Because indirect costs are currently a substantial part of the total health care costs associated with DD, it will be of great interest to see if new treatment strategies reduce the indirect costs of treatment.

The findings of this study will provide a valuable point of reference when new treatment strategies for DD are introduced. For example, surgeons’ preference to use less invasive techniques in older patients may be related to a higher acceptability of the risk of recurrence in these patients when weighed against patient burden and time to recovery. These concerns may become less salient if new treatments result in reduced patient burden and shortened time to recovery. The findings of this study may also inform decisions regarding the distribution of additional research resources to the treatment of DD.

## Electronic supplementary material

Below is the link to the electronic supplementary material.

ESM 1 (PDF 292 kb)
