# Supplementary material for: Current trends in the surgical management of Dupuytren’s disease in Europe: the surgeon’s perspective
Source: Eur Orthop Traumatol. 2012 Mar 2;3(1):25–30. doi: 10.1007/s12570-012-0091-0 (PMC3337993; doi:10.1007/s12570-012-0091-0)
Supplement: Supplementary file 1 — (PDF 292 kb) [file 12570_2012_91_MOESM1_ESM.pdf]

Supplementary material

Article title: **Current trends in the surgical management of Dupuytren's disease in Europe: the surgeon's perspective**

Journal: EOTR

Lars B. Dahlin • Christopher Bainbridge • Piotr Szczypa • Joseph C. Cappelleri • Daniel Guérin • Robert A. Gerber

L. B. Dahlin (✉)

Department of Clinical Sciences - Hand Surgery

Lund University

Skåne University Hospital Malmö

SE-205 02 Malmö, Sweden

e-mail: Lars.Dahlin@med.lu.se

Phone: +46-40-331724

Fax: +46-40-928855

C. Bainbridge

Pulvertaft Hand Centre, Derby, Derbyshire, UK

P. Szczypa

Medical Affairs, Pfizer Ltd., Tadworth, Surrey, UK

J. C. Cappelleri • R. A. Gerber

Medicines Development Group, Pfizer Inc, Groton, CT, USA

D. Guérin

A+A Healthcare Research, Lyon, France

Questionnaire items

Throughout the questionnaire, the term “Dupuytren’s contracture patients” refers to those seen personally by the physician in the last year.

Q0. For a Dupuytren’s patient with any degree of finger contracture/flexion (nodules EXCLUDED), which of the following do you perform? (Responses: Yes/No for each procedure type: Needle fasciotomy/aponeurotomy, Fasciotomy / aponeurotomy [subcutaneous or open], Fasciectomy/ Aponeurectomy, Dermofasciectomy)

Q1. Are your patients on a waiting list for the following procedures? (Response: Yes/No; asked for each procedure type performed by this respondent)

Q2. On average, how long do your patients spend on a waiting list for the following procedures? (Response: average time spent on waiting list awaiting procedure in weeks; asked for each procedure type performed by this respondent)

Q3. On average, how long do you recommend your patients stay off work following procedures below. Please, answer this question for the patient’s first procedure and a re-intervention procedure for recurrence (if any). (Responses: Average time spent out of work following the patient’s first procedure in weeks; Average time spent out of work following a re-intervention procedure in weeks; asked for each procedure type performed by this respondent)

Asked for each procedure type:

Q4. Please indicate below for each of the following elements if it increases or decreases you intention to use a **needle fasciotomy procedure** for your Dupuytren's contracture patients with a finger flexion (excluding nodules only)?

Please use a scale from 1 to 7 where 1 means that the element strongly decreases your intention to use a needle fasciotomy/aponeuortomoy procedure and 7 means that the element strongly increases your intention to use a needle fasciotomy/aponeurotomoy procedure.

Scale

1 Strongly decreases

2 Decreases

3 Slightly decreases

4 Neither decreases nor increases

5 Slightly increases

6 Increases

7 Strongly increases

		Strongly decreases my intention to use a needle fasciotomy procedure					Strongly increases my intention to use a needle fasciotomy procedure
1	Age: below 45	1	2	3	4	5	6	7
2	Age: above 70	1	2	3	4	5	6	7
3	Family history	1	2	3	4	5	6	7
4	Patient's job/profession/way of life requires manual dexterity	1	2	3	4	5	6	7
5	Patient request based on aesthetic reasons	1	2	3	4	5	6	7
6	Patient request based on impairment of activities of daily living (e.g., shaking hands, opening a jar)	1	2	3	4	5	6	7
7	MCP flexion less than 20 degrees	1	2	3	4	5	6	7
8	MCP flexion between 21 and 45 degrees	1	2	3	4	5	6	7
9	MCP flexion greater than 45 degrees	1	2	3	4	5	6	7

10	PIP flexion less than 20 degrees	1	2	3	4	5	6	7
11	PIP flexion between 21 and 45 degrees	1	2	3	4	5	6	7
12	PIP flexion greater than 45 degrees	1	2	3	4	5	6	7
13	Speed of progression of disease over time	1	2	3	4	5	6	7
14	Patient presenting with recurrence	1	2	3	4	5	6	7
15	Patient comorbidities/other risk factors	1	2	3	4	5	6	7
16	Number of fingers involved	1	2	3	4	5	6	7
17	Anticipated long duration of post-surgical physiotherapy							
18	High expectations of success following surgical procedure	1	2	3	4	5	6	7
19	Moderate expectations of success following surgical procedure	1	2	3	4	5	6	7
20	Low expectations of success following surgical procedure	1	2	3	4	5	6	7
21	Others factors, please specify........... ________________________________	1	2	3	4	5	6	7

Asked for each procedure type performed by this respondent:

Q5. Could you rate the **Needle fasciotomy/aponeurotomy** in terms of **satisfaction**, for each of the following attributes (lines)?

Please use a scale from 1 to 7 where 1 means you are not at all satisfied with the criteria and 7 means you are very satisfied with the criteria.

Scale:

1 Totally dissatisfied

2 Dissatisfied

3 Slightly dissatisfied

4 Neither satisfied nor dissatisfied

5 Slightly satisfied

6 Satisfied

7 Very satisfied

		**Needle fasciotomy/ aponeurotomy**
1	Overall satisfaction	
2	Patient satisfaction	

3	Ability to restore a satisfactory degree of finger functionality/range of motion	
4	Patient burden in relation to the treatment (pain, length of recovery...)	
5	Complications associated with the procedure	
6	Likelihood of disease recurrence following the treatment	
7	Time-to-recurrence following the treatment	
8	Time required from start of treatment to completion of recovery	

Q6. Out of 10 Dupuytren's contracture patients with a finger flexion (excluding nodules only) undergoing each of the following treatments **for the patient's first procedure**, how many of them receive **physical therapy** *(including dynamic splint)?* (Response: Number; asked for each procedure type performed by this respondent)

Q7. If Q6>0 patients: On average, how long is the physical therapy *(including dynamic splint)* for each of the treatment mentioned for the patient's first procedure? (Response: Number of weeks or months; asked for each procedure type performed by this respondent)

Q8. If Q6>0 patients: How frequent are the visits for physical therapy *(including dynamic splint)* for the patient's first procedure? (Response options: More than once a week, Every

week, Twice a month, Once a month, or Less than once a month; asked for each procedure type performed by this respondent)

Q9. If Q6>0 patients: How soon is the first follow up appointment after surgery? (Response: Number of weeks; asked for each procedure type performed by this respondent)

Q10. Do you have a specific physical therapy and/or a rehabilitation protocol in your practice for patients undergoing a procedure for their Dupuytren's Contracture? (Response: Yes/No)

Q11. Out of 10 patients that you are treating with a procedure for Dupuytren's contracture, how many of them have you enrolled in this physical therapy protocol? (Response: Number)

Q13. In your practice, what is the average time you require to conduct each procedure? (Response: Average time you take to conduct the following procedure in hours and minutes; asked for each procedure type performed by this respondent)

Now, we will consider the management of recurrence in Dupuytren's contracture.

Please take into account the following definition for "recurrent contracture": "A contracture which is increasing or has returned in a previously treated finger"

Q14. Following the procedure, what percentage of patients has a recurrence of flexion on the same finger (on average)? (Response: Percentage [on average]; asked for each procedure type performed by this respondent)

Q15. Following the procedure, when there is a recurrence, how long after the procedure does it occur (on average)? (Response: Number of months or years [on average]; asked for each procedure type performed by this respondent)
